# Gaucher disease: clinical phenotypes and refining *GBA* mutational spectrum in Thai patients

**DOI:** 10.1186/s13023-021-02151-2

**Published:** 2021-12-20

**Authors:** Tim Phetthong, Thipwimol Tim-Aroon, Arthaporn Khongkraparn, Saisuda Noojarern, Chulaluck Kuptanon, Khunton Wichajarn, Achara Sathienkijkanchai, Kanya Suphapeetiporn, Pimlak Charoenkwan, Adisak Tantiworawit, Naruwan Noentong, Duangrurdee Wattanasirichaigoon

**Affiliations:** 1 Division of Medical Genetics, Department of Pediatrics, Faculty of Medicine Ramathibodi Hospital, Mahidol University, Rama 6 Road, Bangkok, 10400 Thailand; 2 Division of Medical Genetics, Department of Pediatrics, Phramongkutklao Hospital and Phramongkutklao College of Medicine, Bangkok, Thailand; 3 Genetics Section, Department of Pediatrics, Queen Sirikit National Institute of Child Health, Bangkok, Thailand; 4 Department of Pediatrics, College of Medicine, Rangsit University, Bangkok, Thailand; 5 Division of Medical Genetics, Department of Pediatrics, Faculty of Medicine, Khon Kaen University, Khon Kaen, Thailand; 6 Division of Medical Genetics, Department of Pediatrics, Faculty of Medicine Siriraj Hospital, Mahidol University, Bangkok, Thailand; 7 Division of Medical Genetics and Metabolism, Department of Pediatrics, Faculty of Medicine, Chulalongkorn University, Bangkok, Thailand; 8 Division of Hematology and Oncology, Department of Pediatrics, Faculty of Medicine, Chiang Mai University, Chiang Mai, Thailand; 9 Division of Hematology, Department of Internal Medicine, Faculty of Medicine, Chiang Mai University, Chiang Mai, Thailand; 10 Department of Pediatrics, Surin Hospital, Surin, Thailand

**Keywords:** Asian, *GBA*, *GBAP*, L444P, p.L483P, Recombinant allele, Rec1a, Prevalence

## Abstract

**Background:**

Gaucher disease (GD) is a rare lysosomal storage disorder, characterized by hepatosplenomegaly and pancytopenia, with or without neurologic involvement. The disorder is categorized into three phenotypes: GD type 1 or nonneuronopathic GD; GD type 2 or acute neuronopathic GD; and GD type 3 or chronic neuronopathic GD. The purposes of this study were to describe clinical characteristics of Thai GD in patients diagnosed and/or followed up during 2010–2018 and to perform re-genotyping including analysis of *GBA* recombinant alleles which had not been investigated in Thai patients before.

**Results:**

There were 27 patients from seven medical centers, enrolled in the study. All the cases had pediatric onset. GD3 (44.5%) was the most common phenotype, followed by GD2 (40.7%) and GD1 (14.8%), with one case of neonatal GD. The median age of onset for GD1, GD2, and GD3 was 72, 4 and 12 months, respectively, suggesting relatively earlier onset of GD1 and GD3 in Thai patients. All patients with GD1 and most patients with GD3 received ERT. Four patients with GD3 had ERT followed by HSCT. Patients with GD3 who received no or late ERT showed unfavorable outcomes. We identified 14 variants including two novel (p.S384F and p.W533*) and 12 reported pathogenic variants: p.L483P, p.N409S, p.R159W, p.P305A, p.A175G, p.D448H, p.V414L, IVS2+1G>A, IVS6-1G>C, IVS7+1G>C, IVS9-3C>G, and Rec1a. The p.L483P was the most prevalent allele found in this study, at 66% (33/50 alleles), followed by IVS2+1G>A, Rec1a, and IVS6-1G>C. Twenty-four percent of patients were reassigned with validated genotypes, most of whom (4 of 6) were patients with GD2. The [p.S384F + p.W533*] being compounded with p.L483P, was found in the patient with neonatal GD, suggesting that the p.S384F could potentiate the deleterious effect of the p.W533*, and/or vice versa.

**Conclusions:**

Neuronopathic GD was strikingly prevalent among Thai affected population. Homozygous p.L483P was the most common genotype identified in Thai patients. Recombinant allele Rec1a and splicing mutations were associated with GD2 and severe cases of GD3. Mutation spectrum could be useful for designing stepwise molecular analysis, genetic screenings in population, and new therapeutic research for neuronopathic GD.

**Supplementary Information:**

The online version contains supplementary material available at 10.1186/s13023-021-02151-2.

## Background

Gaucher disease (GD) is a lysosomal storage disorder, caused by deficiency of glucocerebrosidase enzyme, resulting in multi-systemic manifestations including hepatosplenomegaly, pancytopenia, with or without neurologic involvement. The disorder is categorized into three clinical phenotypes [1]. Non-neuronopathic or GD type 1 (GD1; OMIM 230800) has predominant hematologic and visceral organ involvement with additional bone symptoms in some patients. Acute neuronopathic GD type 2 (GD2; OMIM 230900) has onset before age two years and rapid progression leading to death before age four years. Subacute neuronopathic GD type 3 (GD3; OMIM 231000) may have onset before or after two years of age, with later onset and slower progression of neurological symptoms. GD3 can be further divided into 3a: mild visceral symptoms but progressive neurologic decline; 3b: severe visceral involvement; 3c: fibrosis and calcification of aorta and cardiac valves; and Swedish L444P-related Norrbottnian phenotype which is characterized by progressive kyphosis and early onset visceral and neurological manifestations [2]. There is also a very rare distinct subtype, perinatal-lethal form associated with severe hepatosplenomegaly and ichthyosiform, nonimmune hydrops, and early death [1]. Moreover, there was a reported patient of an unclassified subtype of Gaucher disease, who presented with a neonatal-onset of hepatosplenomegaly and pancytopenia, without profound neurological disease [3].

GD1 accounts for 95% of GD found among Caucasian population while GD2 and GD3 are more prevalent among Asian affected populations [1, 4–7].

The glucocerebrosidase enzyme is encoded by acid-beta glucosidase (*GBA*) gene located on chromosome 1q21 and spanning 7 kb with 11 coding exons. *GBAP*, a non-functional pseudogene of *GBA*, shares 96% homology of the overall sequence and is located 16 kb downstream [8]. The presence of *GBAP* complicates mutation analysis of *GBA* and can lead to recombinant allele which is much more difficult to detect [6–9].

There have been > 449 damaging *GBA* variants identified, including 357 missense/nonsense/splicing variants and 23 complex rearrangement (the Human Gene Mutation Database, http://www.biobase-international.com). Most *GBA* mutations resulting in GD are point mutations with majority clustering within exons 8–11. N370S (p.Asn409Ser) and L444P (p.Leu483Pro) are the most prevalent variants described among Jewish and non-Jewish affected populations, respectively.

The complex rearrangement is resulted from homologous recombination between the *GBA* and *GBAP*. It could be either non-reciprocal (gene conversion) or reciprocal recombination (gene fusion and partial gene duplication). These alleles are likely underrepresented because it is difficult to analyze and often left out from the routine analysis due to its complexity. It has been shown that recombination accounts for up to 24% in some affected populations, especially in neuronopathic GD [5, 7, 10] and low at 3% in some others [11, 12].

Among GD with complex mutations, gene conversion represents 58% of the recombination. A specific allele, namely Rec1a, is the most prevalent (91%) variant amongst the gene conversion alleles identified [7, 8]. Rec1a has been previously reported in Asian GD [4, 7, 8, 13, 14] but never been detected in Thai patients [15–17]. The possible reason that Rec1a had not been reported in Thai patients, likely because the complex rearrangement/conversion was not detected by the methods used in those studies.

To date, reports of GD with mutation data from Thailand are limited. We previously described four patients with neuronopathic GD and their molecular defects. Herein, we present 27 unreported patients and their genotypes including the first study of recombinant alleles in Thai GD.

## Methods

### Patients

This is a cross-sectional and descriptive study of seven major teaching hospitals: Ramathibodi Hospital, Queen Sirikit National Institute of Child Health, Srinagarind Hospital (Khon Kaen University), Siriraj Hospital, King Chulalongkorn Memorial Hospital, Chiang Mai University Hospital, and Surin Hospital. These institutes cover most of the patients diagnosed with GD in the country. Patients with GD who were newly diagnosed and/or followed during 2010 and 2018 (9 years) were enrolled in the study.

Clinical data including age at onset, age at diagnosis, clinical manifestations, type of GD, enzyme activity, specific treatment received including enzyme replacement therapy (ERT) and hematopoietic stem cell transplantation (HSCT) were retrospectively reviewed. Type of GD was categorized by the treating physicians, according to clinical ground, age of onset, and the presence of neurological involvement. The neurological manifestations referred to bulbar signs (stridor, strabismus, feeding difficulty), pyramidal signs (opisthotonus, spasticity, trismus) and oculomotor apraxia (failure of saccadic eye movement) [1].

GD1 is assigned for patients with the absence of neurological symptoms related to GD throughout the time course. The presence of spasticity, seizure, and/or developmental regression before 2 years of age was assigned as GD2. Patients with any neurological manifestation and/or the ocular signs other than those of GD2 would be categorized as GD3.

### Re-genotyping of the GBA gene and analysis of recombinant alleles

Patient’s mutation data prior to this study were collected, and later compared with the result from the re-genotyping. Archived/left over specimens were sent to Ramathibodi Hospital for re-genotyping. We used amino acid numbering system following Human Genome Variation Society (https://www.hgvs.org/) nomenclature with preceding p., and also provided traditional number without the proceeding p., for example p.L483P and L444P representing the same amino acid variant.

To avoid amplification of non-functional pseudogene, *GBAP*, a nested PCR approach was employed to selectively amplify the *GBA* gene, following established protocols [6, 9]. In brief, the first amplification (10 cycles) using specific primers sets (GBA-f and MTX1P-r) resulted in DNA fragment containing the entire *GBA* gene and part of *MTX1P* (pseudo-*MTX1* gene)*.* This was followed by nested PCR (35 cycles) to amplify the entire *GBA* gene, excluding the *MTX1P*, using different primer pair (GBA-f and GBA-r; Additional file 1: Table S1 and Figure S1A). Both PCR reactions shared similar constituents as follow: 25 ul of total volume, 250 ng of genomic DNA, 2.5 µM of each primer, 1 × PCR buffer, 2 mM MgCl_2_, and 2.5 U of Taq polymerase. (TaKaRa LA Taq™). The PCR cycling conditions were 95 °C, 1 min; then 10 (or 35) cycles of denaturation at 95 °C, 30 s and annealing at 60 °C, 30 s with extension at 72 °C, 5 min; then final extension at 68 °C for 10 min.

The expected final 6.5 kb-PCR product was then subjected to Sanger sequencing of all 11 *GBA* exons, using ABI 3730xl DNA Analyzer (Macrogen®, Korea). Details of sequencing primers were provided in the Supplementary data (Additional file 1: Figure S1B and Table S1). Gene conversion allele could be detected by careful examination of the sequence, focusing on every site known to be different between the *GBA* and *GBAP* (Additional file 1: Figure S2). The presence of three or more point mutations in *cis*, distinctive sequence of *GBAP*, and/or detection of the cross over site between *GBA* and *GBAP* (as a result of *GBAP* being converted into the *GBA* gene) would indicate gene conversion. The assignments of gene conversion allele were as follows: Rec1a − [L444P + A456P + V460V]; Rec2a −  [D409H + L444P + A456P + V460V]; Rec3a −  [55-bp deletion in exon 9 + D409H + L444P + A456P + V460V], as previously established [6–9, 18]. As for Rec4a, Rec5a, and Rec8a, the final PCR product’s size would be several-hundred-bp-smaller than 6.5 kb which could be seen on the agarose gel, in addition to the [55-bp deletion in exon 9 + D409H + L444P + A456P + V460V] plus the distinctive variants of *GBAP* (Additional file 1: Figure S2). Rec6a and Rec7a alleles could not be detected by the method used in this study.

The GenBank reference sequences used were ENST00000368373.8 and NM_000157.4. Variant pathogenicity was determined in accordance with the guidelines of the American College of Medical Genetics and Genomics (ACMG) and the Association for Molecular Pathology (AMP) [19]. Rec1a positive control was kindly given by Prof. Yin-Hsiu Chien, National Taiwan University Hospital.

## Results

### Patients’ demographic and clinical data

There were 27 patients (12 male and 15 female) enrolled in the study, including 4 GD1, 10 GD2,12 GD3, and 1 neonatal GD. To the best of our knowledge, this report covers about 80% of GD patients in Thailand during the time of study. Eleven of 27 (40.7%) patients were recruited from Ramathibodi Hospital and the remainders (59.3%) were enrolled via the other six participating hospitals (3 in the Central region, 1 in the Northern and 2 in the Northereastern area). Of 27 patients, 10 originated from the Central region, 10 from the Northern area, and the other 7 from Northeastern region.

The age at onset ranged from 2 weeks (wk) to 10 years (y), and age at diagnosis ranged between 4 months (m) and 26 y 11 m (Tables 1, 2). The median and mean age at onset, at diagnosis, and time to diagnosis for each clinical type was as detailed in Table 1.

**Table 1:** Demographic data of Gaucher disease patients in the present study (n = 27)

Chracteristics	GD1 (N = 4)	GD2 and neonatal GD (N = 11)	GD3 (N = 12)
Sex			
Male	0	5	7
Female	4	6	5
Age of onset (m.)^a,b^			
Median (range)	72 (14–120)	4 (1–10)	12 (6–36)
Mean (± SD)	68.7 (53.1)	4.3 (2.7)	16.3 (11)
Age at diagnosis (m.)^a^			
Median (range)	72 (18–252)	8 (6–24)	34.5 (11–84)
Mean (± SD)	114 (122.5)	9.7 (5.6)	35.8 (24.5)
Time to diagnosis (m.)^a,b^			
Median (range)	4 (0–132)	4 (1–20)	19 (0–48)
Mean (± SD)	45.3 (75.1)	5.4 (5.9)	19.6 (17.2)

^a^Patients GD-5 (with N370S) and GD-28 (neonatal GD) were excluded from the computation due to being outliners

^b^Patient GD-22 was excluded because of no available data of the age at onset

**Table 2:** Clinical characteristics and specific treatment received of each patient (n = 27)

Patient ID	Sex	Age at onset	Age at Dx	Type of GD	Presentation at Dx	PE at Dx/BMA	Specific treatment	Outcome; age^a^
GD-5	F	8 y	26 y 11 m	1	AD, AN, TN	HSP	ERT	Alive; 34 y
GD-17	F	6 y	6 y	1	AD, AN, TN	HSP	ERT	Alive; 16 y
GD-19	F	10 y	21 y	1	AD, AN, TN, skin bruise	HSP	ERT	Alive; 25 y
GD-23	F	14 m	18 m	1	AD, AN, TN	HSP	ERT	Alive; 17 y
GD-11	F	3 m	8 m	2	AD, GDD, FTT, SZ	HSP, esotropia, SpT/ GC	None	Dead; 1 y 6 m
GD-14	F	4 m	2 y	2	AD, AN, DR, rectal prolapse	HSP, SpT/GC	None	Dead; 2 y 6 m
GD-16	F	5 m	6 m	2	AD, GDD	HSP, SpT	None	Dead; 10 m
GD-18	M	6.5 m	9 m	2	AD, AN, TN, DR	HSP, SpT	None	Dead; 1 y
GD-22	M	NA	9 m	2	AD, GDD	HSP	None	Dead; 1y
GD-25	M	1 m	9 m	2	AD, jaundice, hypertonia	HSP, SpT	None	Dead; 1 y 6 m
GD-27	F	10 m	11 m	2	AD, AN, TN, DR, SZ (status epilepticus)	HSP, esotropia, opisthotonus/GC	None	Dead; 1 y 1 m
GD-29	F	3 m	7 m	2	AD, hypertonia	HSP, OP, opisthotonus	None	Dead; 1–2 y
GD-30	F	4 m	7 m	2	AD, hypertonia	HSP,OP, opisthotonus/ GC	None	Dead; 1–2 y
GD-31	M	2 m	6 m	2	AD, hypertonia	HSP, OP, opisthotonus/GC	None	Dead; 1–2 y
GD-28	M	2 wk	4 m	Neonatal	AD, TN, skin bruise	HSP/GC	ERT	Dead; 2 y
GD-4	M	6 m	1 y 2 m	3b	AD, AN, TN, GDD	HSP, OP, esotropia,/GC	ERT& HSCT	Alive; 16 y 10 m
GD-6	F	9 m	12 m	3b	AD, AN, TN	HSP, OP/GC	ERT& HSCT	Alive; 9 y 11 m
GD-7	F	1 y	2 y 10 m	3b	AD, AN, TN	HSP, OP	ERT& HSCT	Alive; 8 y 2 m
GD-8^b^	M	1 y	3 y	3b	AD, AN, TN	HSP, OP	ERT& HSCT	Alive; 17 y 7 m
GD-9^b^	M	8 m	2 y	3b	AD, AN, TN	HSP	ERT	Alive; 11 y 5 m
GD-10	M	1 y	2 y 11 m	3b	AD, AN, TN	HSP, OP	None	Dead; 2 y 3 m
GD-12	M	3 y	3 y	3b	AD, AN, TN	HSP/ GC	ERT	Alive; 16 y 10 m
GD-13	F	6 m	1 y	3b	AD, AN	HSP, OP/ GC	ERT	Alive; 7 y 9 m
GD-15	M	2 y	6 y	3b	AD	HSP	ERT	Alive; 8 y
GD-20	F	2 y	5 y	3a/b	AD, DR	HSP	None	Dead; ~ 7 y
GD-24	M	10 m	11 m	3a/b	AD, AN, TN	HSP, OP, esotropia	ERT	Alive; 19 y
GD-26	F	3 y	7 y	3a/b	AD, SZ	HSP	ERT	Dead; 13 y

AD, abdominal distention; AN, anemia; BMA, bone marrow aspiration; DR, developmental regression; Dx, diagnosis; F, female; FTT, failure to thrive; GC, Gaucher cell; GDD, global developmental delay; HSP, hepatosplenomegaly; M, male; m, months; mSZ, myoclonic seizures; NA, not available; OP, oculomotor apraxia; PE, physical examination; SpT, spastic tone; SZ, seizures; TN, thrombocytopenia; wk, weeks; y, years

^a^Age at the time of this report, for living patient

^b^Siblings

Abdominal distension is the most consistent finding in all patients (100%), followed by anemia (59.3% or 16/27), thrombocytopenia (55.6% or 15/27), and neurological symptoms including developmental delay/regression with/without seizure disorders (51.9% or 14/27). Oculomotor apraxia (supranuclear gaze palsy) and/or esotropia was noted at the first presentation or during the time course in 44.4% (12/27).

All (4) patients with GD1 received ERT and are still alive. Ten of 12 patients with GD3 received ERT. Four of the 10 patients subsequently underwent HSCT and had ERT discontinued soon after and they were alive at the time of this writing. One patient with GD3 (GD-26) had ERT initiated at 11 years of age, she later developed seizures and neurological deterioration, leading to death at the age of 13 years. Two patients with GD3 were not treated with ERT due to unavailability of the treatment at that time (year 2010). As for GD2, all (10) patients did not receive ERT and died at the age 10 months–2 years 6 months. One patient with neonatal-typed GD was attempted with ERT for 6 months which led to an improvement of hematologic and visceral symptoms, but ongoing neurological decline and eventually death at the age of 2 years. There was no apparent correlation between the residual enzyme activity and type of GD.

As for why some (4) patients underwent HSCT, there were multiple reasons for this. The four patients had GD3 and genotype of homozygous p.L483P, at risk for progressive neurological deterioration. Before the year 2013, ERT was not reimbursable in the country, two patients (GD-4 and GD-6) had received ERT under a compassionate use program with unclear future of a long-term supply. Therefore, HSCT was offered to the patients, following 1–2 years of ERT. After 2013, additional families (GD-7 and GD-8) were interested in having HSCT for a cure instead of life-long ERT and for that it might be helpful in mitigating the progression of neurological manifestations. One patient (GD-4) received HLA-matched unrelated donor transplantaion and the other three patients (GD-6, GD-7, and GD-8) received HLA-haploidentical transplantation. The details of patients’ clinical and laboratory data including outcomes are being prepared for a separate manuscript. It should be mentioned that HSCT for severe thalassemia, a common genetic disorder in Thailand, has been widely accepted in our country which may influence the decision of the families to accept HSCT for GD3.

### GBA mutational spectrum and recombinant allele, Rec1a

Prior to the study, mutation data of the enrolled patients were determined in four different laboratories including three in the country and one abroad. These tests did not cover analysis for recombinant alleles.

**Fig. 1:**
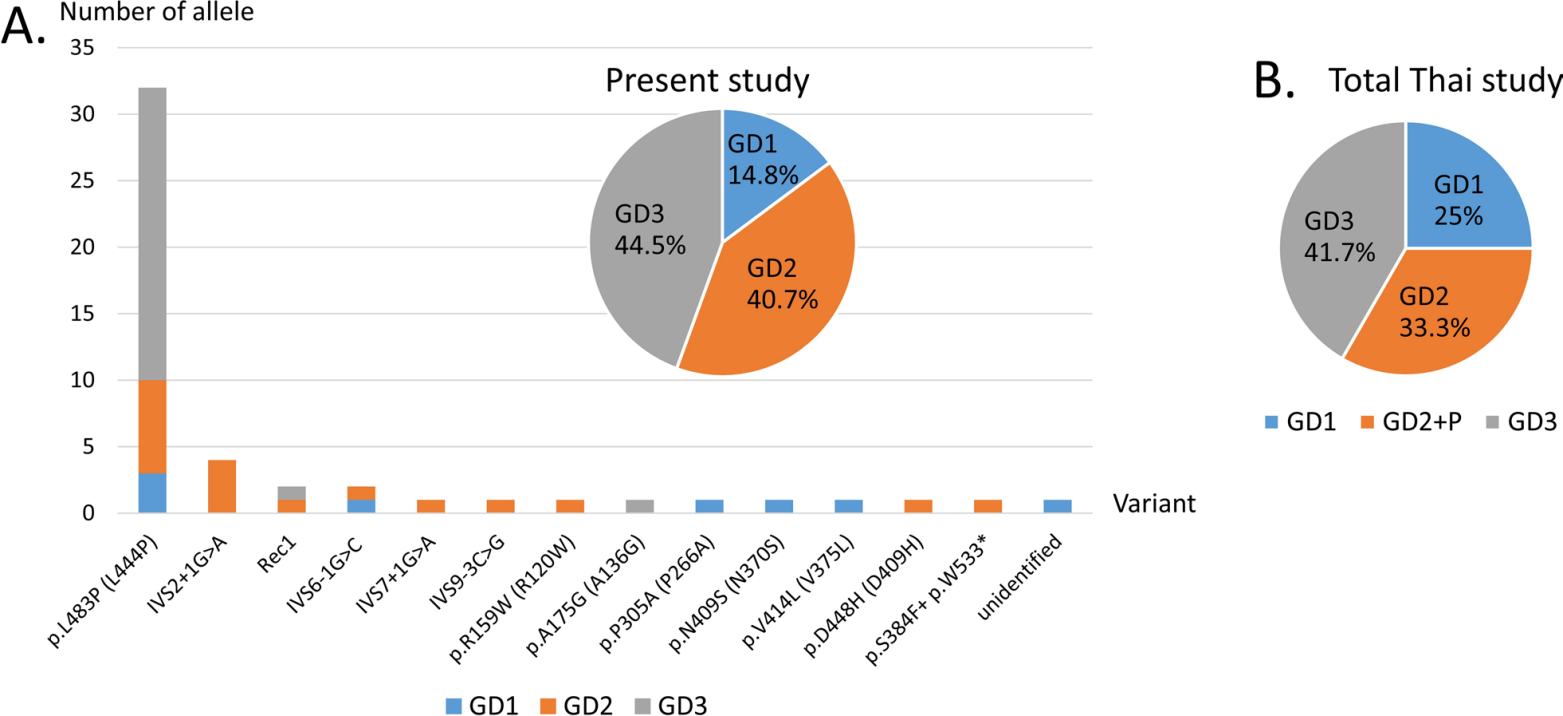
Prevalence of Gaucher disease phenotypes and *GBA* mutant allele in Thai patients. **A** Data of the present cohort (n = 27 for phenotype analysis; 25 individuals of 50 alleles for genotype analysis). **B** Data combined with three previous reports (total n = 36 for phenotype analysis)

The re-genotyping in this study identified 14 variants including two novel (p.S384F and p.W533*) and 12 reported pathogenic variants: p.L483P, p.N409S, p.R159W, p.P305A, p.A175G, p.D448H, p.V414L, IVS2+1G>A, IVS6-1G>C, IVS7+1G>C, IVS9-3C>G, and Rec1a (RecNciI) (Table 3, Fig. 1 and Additional file 1: Figure S3).

**Table 3:** Genotype according to phenotype of each GD patient (n = 27)

Patient ID	GD subtype	Enzyme activity^c^ (%)^h^	Prior genotype		Validated genotype	
			Allele 1	Allele 2	Allele 1	Allele 2
GD-5	1	0.24 (3.1)	p.L483P	p.N409S	Same	
GD-17	1	0.72 (9.3)	p.L483P	p.A175G	p.L483P	p.P305A
GD-19	1	0.67 (8.6)	p.L483P	Unidentified	Same	
GD-23	1	0.96^d^ (13.5)	p.V414L	IVS6−1G>C	Same	
GD-11	2	0.01 (0.1)	p.L483P	IVS2+1G >A	p.L483P	p.R159W
GD-14	2	1.99 (25.6)	IVS2+1G>A	IVS9−3C>G	Same	
GD-16	2	0.01 (0.1)	p.L483P	p.F346L, IVS2+1G>A	p.L483P	IVS2 +1G>A
GD-18	2	0.65 (8.4)	p.L483P	p.L483P	p.L483P	Rec1a
GD-22	2	0.37 (4.8)	p.D448H	IVS2+1G >A	Same	
GD-25	2	0.61^d^ (8.6)	p.L483P	IVS2+1G>A	Same	
GD-27	2	1.06^e^ (12.5)	p.L483P	p.L483P	p.L483P	IVS6−1G>C
GD-29	2	0.2^ g^ (16.7)	p.L483P	IVS7+1G>A	ND	
GD-30	2	NA	ND	ND		
GD-31	2	NA	ND	ND		
GD-28	Neonatal	1.05^f^ (13.2)	p.L483P	p.S384F + p.W533*	Same	
GD-4	3b	0.32 (4.1)	p.L483P	p.L483P	Same	
GD-6	3b	0.65 (8.4)	p.L483P	p.L483P	Same	
GD-7	3b	0.12 (1.5)	p.L483P	p.L483P	Same	
GD-8^a^	3b	0.58 (7.5)	p.L483P	p.L483P	Same	
GD-9^b^	3b	0.17 (2.2)	p.L483P	p.L483P	Same	
GD-10	3b	1.19 (15.3)	p.L483P	p.L483P	Same	
GD-12	3b	0.47 (6.0)	p.L483P	p.L483P	Same	
GD-13	3b	0.58 (7.5)	p.L483P	p.L483P	Same	
GD-15	3b	1.74 (22.4)	p.L483P	p.L483P	Same	
GD-20	3a/b	NA	p.L483P	p.A175G	p.A175G	Rec1a
GD-24	3a/b	1.21^d^ (17.0)	p.L483P	p.L483P	Same	
GD-26	3a/b	1.11^d^ (15.6)	p.L483P	p.L483P	Same	

NA, data not available; ND, not done

^a,b^Siblings

^c^Glucocerebrosidase enzyme activity in leukocytes with normal range 7.77–11.53 nmol/h/mg protein

^d^7.1–16.9

^e^8.48–13.52

^f^7.96–17.89

^g^> 1.2, According to the responsible laboratories

^h^Percentage of the subject’s glucocerebrosidase enzyme activity compared to the laboratory's lower limit of normal

The p.S384F was classified as likely pathogenic (PM1, PM2, PP2, PP5) and the p.W533* as pathogenic (PVS1, PM2, PP3), according to the ACMG/AMP guideline. These two variants were not found in the gnomAD database (MAF = 0) and Thai Reference Exome Database (MAF 0/2000; T-REx, https://trex.nbt.or.th/). Multiple lines of computational evidence (DEOGEN2, FATHMM-MKL, M-CAP and MutationTaster) suggested pathologic impact of the variants. The p.S384F and p.W533* were submitted to ClinVar Database and were assigned references#, VCV000917863 and VCV000917862, respectively (https://www.ncbi.nlm.nih.gov/clinvar/). Interestingly, the two variants were present in *cis* [p.S384F + p.W533*] and genetically compounded with p.L483P in a single patient with neonatal GD (GD-28; Table 3). The [p.S384F + p.W533*] allele was inherited from the patient’s asymptomatic mother and the p.L483P from the father.

The prevalence of variants identified according to phenotypes was shown in Fig. 1A and Additional file 1: Table S2. Genotype of each patient was summarized in Table 3. Allele frequency of the *GBA* variants in affected populations was compared between this study and other studies (Table 4).

**Table 4:** Frequencies of *GBA* pathogenic variants in Thai Gaucher disease and other ethnicities

Mutant allele	Thai, total^a^ [15–17]	Chinese [13]	Japanese [4]	Korean [6]	Indian [22]	Jewish [11]	Non-Jewish [11]
p.L483P (L444P)	41 (60.3%)	21 (47.7%)	41 (41%)	15 (20.8%)	40 (69%)	6 (3.9%)	29 (39.2%)
IVS2+1G>A	4 (5.8%)	1 (2.3%)	1 (1%)	1 (1.4%)	0 (0%)	4 (2.6%)	1 (1.4%)
Rec1	2 (2.9%)	3 (6.8%)	0 (0%)	2 (2.8%)	0 (0%)	0 (0%)	0 (0%)
IVS6−1G>C	4 (5.8%)	2 (4.5%)	0 (0%)	0 (0%)	0 (0%)	0 (0%)	0 (0%)
IVS9−3C>G	3 (4.4%)	0 (0%)	0 (0%)	0 (0%)	0 (0%)	0 (0%)	0 (0%)
IVS7+1G>A	1 (1.5%)	0 (0%)	0 (0%)	0 (0%)	0 (0%)	0 (0%)	0 (0%)
p.F252I (F213I)	2 (2.9%)	0 (0%)	14 (14%)	9 (12.5%)	0 (0%)	0 (0%)	0 (0%)
p.R159W (R120W)	1 (1.5%)	1 (2.3%)	0 (0%)	0 (0%)	0 (0%)	0 (0%)	0 (0%)
p.A175G (A136G)	1 (1.5%)	0 (0%)	0 (0%)	0 (0%)	0 (0%)	0 (0%)	0 (0%)
p.P305A (P266A)	1 (1.5%)	0 (0%)	0 (0%)	0 (0%)	0 (0%)	0 (0%)	0 (0%)
p.N409S (N370S)	1 (1.5%)	0 (0%)	0 (0%)	0 (0%)	0 (0%)	107 (70.3%)	20 (27%)
p.V414L (V375L)	1 (1.5%)	2 (4.5%)	0 (0%)	0 (0%)	0 (0%)	0 (0%)	0 (0%)
p.D448H (D409H)	1 (1.5%)	2 (4.5%)	5 (5%)	1 (1.4%)	4 (6.9%)	0 (0%)	1 (1.4%)
p.S384F + p.W533*	1 (1.5%)	0 (0%)	0 (0%)	0 (0%)	0 (0%)	0 (0%)	0 (0%)
p.N227K (N188K)	1 (1.5%)	0 (0%)	0 (0%)	0 (0%)	0 (0%)	0 (0%)	0 (0%)
p.Y402H	1 (1.5%)	0 (0%)	0 (0%)	0 (0%)	0 (0%)	0 (0%)	0 (0%)
p.X537A	1 (1.5%)	0 (0%)	0 (0%)	0 (0%)	0 (0%)	0 (0%)	0 (0%)
Others	0 (0%)	12 (27.2%)	23 (23%)	44 (61.1%)	14 (24.1%)	29 (19%)	5 (6.8%)
Unidentified	2 (2.9%)	0 (0%)	16 (16%)	0 (0%)	0 (0%)	6 (4%)	18 (24.3%)
Total	68 (100%)	44 (100%)	100 (100%)	72 (100%)	58 (100%)	152 (100%)	74 (100%)

^a^Data from the present study plus those from Chavananon et al. [15]; Suwannarat et al. [16] and Tammachote et al. [17]

## Discussion

To our knowledge, this the first multicenter study and analysis of *GBA* recombinant allele in Thai patients, representing the landscape of GD in Thailand in recent years. In the present cohort, all the cases had pediatric onset. GD3 (44.5%) was the most common phenotype in the Thai population, followed by GD2 (40.7%) and GD1 (14.8%), making neuronopathic GD accounting for the majority of cases. When combining our data to previous reports with available information on types of GD and mutations, it still supported that GD3 was the most prevalent phenotype (41.7%) in Thai patients, followed by GD2 (33.3%), and higher proportion of GD1 (25.0%) (Fig. 1B and Additional file 1: Table S2) [15–17].

Notably, our patient GD-28 had neonatal-onset hepatosplenomegaly, anemia, and thrombocytopenia and skin bruises. There was no evidence of neurological disease indicating GD2 or any ichthyosiform or collodion skin abnormalities found in perinatal-lethal form GD. We performed literature serach and found only one patient who was described with homozygous p.D448H and the phenotypes similar to our patient [3].

In the present cohort, 85.2% (23/27) of the patients developed first symptoms at age ≤ 5 years and 74.1% (20/27) at age ≤ 1 year of age, indicating relatively early and severe phenotype of Thai GD as compared to those of Western population. All GD3 patients manifested their first symptoms at age ≤ 3 years and two-thirds at age ≤ 1 year. Nine of 12 GD3 patients had subtype 3b, while the others had phenotypes overlapping between 3a and 3b. Patients with GD3 who did not receive ERT died at very young age while those with comparable onset who had ERT were still living. Early diagnosis and timely initiation of ERT appeared to result in clinical improvement and slowing down and/or preventing the progression of the neurological manifestations. Delayed diagnosis and treatment with ERT led to inevitable neurological progression and death, as seen in one of our patients (GD-26).

The median age of onset of GD1 and GD3 was at 72 and 12 months, respectively, indicating earlier age of onset as compared to Western affected population. As for, GD2, the onset of symptom was at 4.0 months and natural course did not seem to be different from those described in the literature [1]. The average time to diagnosis was shortest in GD2, at 5.4 months, and longer in GD3 at 19.6 months., and GD1 at 45.3 months. This likely reflected the nature of rapid disease progression of GD2, demanding for immediate investigation.

The p.L483P was the most prevalent allele found in this study, at 66% (33/50 alleles), followed by IVS2+1G>A (8%), Rec1a (4%), and IVS6-1G>C (4%) (Table 2). These four alleles together accounted for 82% (41/50) of the mutant alleles identified in this cohort or 75% (51/68) of total Thai GD alleles (Additional file 1: Figure S2) [15–17].

Rec1a allele was found in two patients with neuronopathic GD in the present study, supporting its association with severe disease. Rec1a has been described to be associated with poor clinical outcomes and sometimes mistaken as p.L483P (L444P) [7, 8]. Previous studies have shown that a significant number of GD patients were initially assigned with point mutation(s), but in fact they carried recombinant allele(s) [6–9]. All other variants were found, each in one patient. The alleles associated with GD2 in the present study included p.R159W, p.D488H, p.L483P, Rec1a, IVS2+1G>A, IVS6-1G>C, IVS7+1G>A, and IVS9-3C>G.

In this cohort, the most common genotype was homozygous p.L483P, found in 11 of 12 individuals with GD3, suggesting more homogeneous population of GD3 in Thai affected individuals. Of the 11 patients with homozygous p.L483P, 5 were from the Northern area, 4 from the Northeastern area, and 2 from the Central region, implying a nationwide distribution of this genotype. The second common genotype was genetic compound between p.L483P and other allele which was found in 11 patients: 1 GD3, 6 GD2, 1 neonatal GD, and 3 GD1. We observed that genetic compounds between p.L483P and the recombinant allele, splicing variants, or known severe missense alleles (p.D488H and p.R159W) would lead to neuronopathic GD while the genetic compounds between p.L483P and p.N409S or p.P305A would result in GD1 with slow clinical progression, as described previously [20]

Twenty-four percent (6 of 25 studied) of patients in this cohort were reassigned with validated genotypes, most of whom (4 of 6) were patients with GD2. Therefore, we suggest inclusion of recombinant allele analysis for Thai GD patients especially those with suspected GD2 and GD3 with rapid progression. Plausible explanations for the discrepancy between the results from the re-genotyping and the prior mutations were using different detailed method of mutation screening, such as using *vs* not using long ranged PCR, excluding *vs* not excluding the pseudogene, and limited mutation screening (only for common mutations). Our data support previous observation that the detection of recombinant allele and correct variants of *GBA* depends on the methodology of genetic analysis [12]. The correct identification of mutant alleles could lead to accurate genetic counseling, reproductive planning, and therapeutic decision making.

As for why mutations in some patients (GD-11, GD-17, and GD-27) were drastically different from its initial reports, our guess was that the orginal sequencing was just incorrect, as the method used for the *GBA* analysis did not excluded the GBA pseudogene (*GBAP*). The IVS2+1G>A is an allele present in the *GBAP* (Additional file 1: Figure S2), while it could be a pathogenic mutation present in the *GBA* gene of some patients. The p.R159W is a known pathogenic variant associated with severe phenotypes of GD. The p.A175G and p.L483P represent common alleles in *GBAP* (Additional file 1: Figure S2) whereas the p.P305A and c.IVS6-1G>C are not of the *GBAP* and have been described as pathogenic variants in GD.

Given the [p.S384F + p.W533*] being compounded with p.L483P, resulted in an extreme phenotype of neonatal GD, we hypothesized that the p.S384F could potentiate the deleterious effect of the nonsense mutation, p.W533*, and/or vice versa. Further investigations are required to confirm the pathogenic mechanism of these intriguing alleles. The presence of two alleles in *cis* has been shown associated with a more severe phenotype in GD, homozygous D409H leading to GD3 with cardiac phenotype in most cases whilst homozygous [D409H + H255Q] resulting in GD2 [21].

Our result suggested that mutation spectrum of *GBA* in Thai patients was more homogeneous than ever believed and was different from those seen in Caucasian populations, and more similar to those of East Asian descendants [4, 6, 11, 13, 15–17, 22].

The older report of GD in Thailand, during 1966–1998, consisting of 25 GD patients diagnosed over 33 year-period or 0.8 case per year [23], When combining our data with other recent reports of Thai GD, a total number of patients was 33 (27 + 5 + 1) over the 9 years of study (2010–2018) or 3.7 new cases per year [15, 17]. These data could reflect the better awareness of GD among Thai physicians, resource availability including clinical geneticists, diagnostic facilities, and reimbursement for ERT (since 2013) and HSCT (since 2015), which have been escalating lately. We estimate the prevalence of GD in the Thai population at least 1 in 156,818 and carrier frequency of 1 in 198 as ascertained by using Hardy–Weinberg equation and 6,449,073 live births during the study years, and based on the assupmption that our study population accounts for 80% of total patients across the country (http://statbbi.nso.go.th/staticreport). We believe that these numbers could be rather underestimated due to some hospitals not included in the study and patients escaping diagnosis especially those with later onset and mild GD (possible GD1).

As it is known that ERT does not cross blood brain barrier and that patients with GD3 could exhibit more advanced neurological manifestation some time in their life; therefore, new therapeutic approach and/or adjunct treatment targeting to protect neurological involvement may be required to overcome these challenges. Long-term outcome of GD3 with quite homogeneous genotype of homozygous p.L483P deserves further investigation..

There are a number of limitations of this work including missing data due to the natures of the retrospective study and varying experiences of clinicians in detecting specific subtle signs (i.e. oculomotor apraxia) which may perturb the accuracy of clinical data collected and the assignment of GD phenotype in particular GD1 and GD3. Lastly, analysis for reciprocal recombinant alleles and some gene conversion alleles was not included in this study, which might affect the variant distribution, though the effect was believed to be small due to its rarity.

## Conclusion

GD3 followed by GD2 was strikingly prevalent among the Thai affected population. Homozygous p.L483P was the most common genotype identified in Thai GD. Recombinant allele Rec1a and splicing mutations were associated with GD2 and severe cases of GD3. Mutation spectrum and its frequency could be useful for designing stepwise molecular analysis, genetic screenings in population, and new therapeutic research for neuronopathic GD.

## Supplementary Information

**Additional file 1.** Supplemental methods and results.

## Data Availability

Additional data are provided in Supplementary materials and ClinVar Database (https://www.ncbi.nlm.nih.gov/clinvar/).

## Abbreviations

ERT: Enzyme replacement therapy
*GBA*: Acid-beta glucosidase gene
*GBAP*: Acid-beta glucosidase pseudogene gene
GD: Gaucher disease
GD1: Gaucher disease type 1
GD2: Gaucher disease type 2
GD3: Gaucher disease type 3
HSCT: Hematopoietic stem cell transplantation

## References

[1] Pastores GM, Hughes DA. Gaucher Disease. In: Adam MP, Ardinger HH, Pagon RA, Wallace SE, Bean LJH, Mirzaa G, et al., editors. GeneReviews((R)). Seattle (WA). 1993. 20301446

[2] Germain DP (2004). Gaucher's disease: a paradigm for interventional genetics. Clin Genet.

[3] Roth P, Sklower Brooks S, Potaznik D, Cooma R, Sahdev S (2005). Neonatal Gaucher disease presenting as persistent thrombocytopenia. J Perinatol.

[4] Eto Y, Ida H (1999). Clinical and molecular characteristics of Japanese Gaucher disease. Neurochem Res.

[5] Ida H, Iwasawa K, Kawame H, Rennert OM, Maekawa K, Eto Y (1995). Characteristics of gene mutations among 32 unrelated Japanese Gaucher disease patients: absence of the common Jewish 84GG and 1226G mutations. Hum Genet.

[6] Jeong SY, Park SJ, Kim HJ (2011). Clinical and genetic characteristics of Korean patients with Gaucher disease. Blood Cells Mol Dis.

[7] Wan L, Hsu CM, Tsai CH, Lee CC, Hwu WL, Tsai FJ (2006). Mutation analysis of Gaucher disease patients in Taiwan: high prevalence of the RecNciI and L444P mutations. Blood Cells Mol Dis.

[8] Tayebi N, Stubblefield BK, Park JK, Orvisky E, Walker JM, LaMarca ME (2003). Reciprocal and nonreciprocal recombination at the glucocerebrosidase gene region: implications for complexity in Gaucher disease. Am J Hum Genet.

[9] Jeong SY, Kim SJ, Yang JA, Hong JH, Lee SJ, Kim HJ (2011). Identification of a novel recombinant mutation in Korean patients with Gaucher disease using a long-range PCR approach. J Hum Genet.

[10] Koprivica V, Stone DL, Park JK, Callahan M, Frisch A, Cohen IJ (2000). Analysis and classification of 304 mutant alleles in patients with type 1 and type 3 Gaucher disease. Am J Hum Genet.

[11] Horowitz M, Pasmanik-Chor M, Borochowitz Z, Falik-Zaccai T, Heldmann K, Carmi R (1998). Prevalence of glucocerebrosidase mutations in the Israeli Ashkenazi Jewish population. Hum Mutat.

[12] Tajima A, Yokoi T, Ariga M, Ito T, Kaneshiro E, Eto Y (2009). Clinical and genetic study of Japanese patients with type 3 Gaucher disease. Mol Genet Metab.

[13] Feng Y, Huang Y, Tang C, Hu H, Zhao X, Sheng H (2018). Clinical and molecular characteristics of patients with Gaucher disease in Southern China. Blood Cells Mol Dis.

[14] Zhang W, Oehrle M, Prada CE, Schwartz IVD, Chutipongtanate S, Wattanasirichaigoon D (2017). A convenient approach to facilitate monitoring Gaucher disease progression and therapeutic response. Analyst.

[15] Chavananon S, Sripornsawan P, Songthawee N, Chotsampancharoen T (2021). Successful treatment of gaucher disease with matched sibling hematopoietic stem cell transplantation: a case report and literature review. J Pediatr Hematol Oncol.

[16] Suwannarat P, Keeratichamroen S, Wattanasirichaigoon D, Ngiwsara L, Cairns JR, Svasti J (2007). Molecular characterization of type 3 (neuronopathic) Gaucher disease in Thai patients. Blood Cells Mol Dis.

[17] Tammachote R, Tongkobpetch S, Srichomthong C, Phipatthanananti K, Pungkanon S, Wattanasirichaigoon D (2013). A common and two novel GBA mutations in Thai patients with Gaucher disease. J Hum Genet.

[18] Hruska KS, LaMarca ME, Scott CR, Sidransky E (2008). Gaucher disease: mutation and polymorphism spectrum in the glucocerebrosidase gene (GBA). Hum Mutat.

[19] Richards S, Aziz N, Bale S, Bick D, Das S, Gastier-Foster J (2015). Standards and guidelines for the interpretation of sequence variants: a joint consensus recommendation of the American College of Medical Genetics and Genomics and the Association for Molecular Pathology. Genet Med.

[20] Filocamo M, Mazzotti R, Stroppiano M, Seri M, Giona F, Parenti G (2002). Analysis of the glucocerebrosidase gene and mutation profile in 144 Italian gaucher patients. Hum Mutat.

[21] Michelakakis H, Moraitou M, Dimitriou E, Santamaria R, Sanchez G, Gort L (2006). Homozygosity for the double D409H+H255Q allele in type II Gaucher disease. J Inherit Metab Dis.

[22] Barney AM, Danda S, Abraham A, Fouzia NA, Gowdra A, Abraham SSC (2021). Clinicogenetic profile, treatment modalities, and mortality predictors of gaucher disease: a 15-year retrospective study. Public Health Genomics.

[23] Tanphaichitr VS, Suvatte V, Mahasandana C, Sachapong P, Veerakul G, Kankirawatana S (1999). Gaucher's disease;thirty-two years experience at Siriraj Hospital. Southeast Asian J Trop Med Public Health.

