# Supplementary material for: Gaucher disease: clinical phenotypes and refining GBA mutational spectrum in Thai patients
Source: Orphanet J Rare Dis. 2021 Dec 20;16:519. doi: 10.1186/s13023-021-02151-2 (PMC8686639; doi:10.1186/s13023-021-02151-2)
Supplement: Supplementary file 1 — Additional file 1. Supplemental methods and results. [file 13023_2021_2151_MOESM1_ESM.docx]

**Supplementary data**

**Table S1** Sequences of primers used in this study [9]

| Name | Direction | Sequences | | Position^a^ |
| --- | --- | --- | --- | --- |
| ***PCR primers*** | | |  |  |
| GBA-f | Forward | 5′-TCCTAAAGTTGTCACCCATACATG-3′ | | chr1:155,211,182-155,211,206 |
| MTX1P-r | Reverse | 5′-CCAACCTTTCTTCCTTCTTCTCAA-3′ | | chr1:155,202,291-155,202,315 |
| GBA-r | Reverse | 5′-TAGTCACAGACAGCGTGTGAGC-3′ | | chr1:155,204,705-155,204,727 |
| ***Sequencing primers*** | | |  |  |
| GBA-F1 | Forward | 5'-GGAAGCCAATTGCCCTTTAG-3' | | chr1:155,211,119-155,211,139 |
| GBA-F2 | Forward | 5'-CTGTGGGCCTTGTCCTAATG-3' | | chr1:155,210,619-155,210,639 |
| GBA-F3 | Forward | 5'-CCAAGGGGTGAGGAATTTTG-3' | | chr1:155,209,954-155,209,974 |
| GBA-F4 | Forward | 5'-CCCATCCAGGCTAATCACAC-3' | | chr1:155,209,684-155,209,704 |
| GBA-F5 | Forward | 5'-ACAGGTTCCAACCCAGGAG-3' | | chr1:155,208,194-155,208,214 |
| GBA-F6 | Forward | 5'-GGAGGCTAATGGCTGAACC-3' | | chr1:155,207,856-155,208,876 |
| GBA-F7 | Forward | 5'-AGGCTGTTCTCGAACTCCTG-3' | | chr1:155,207,011-155,207,031 |
| GBA-F8 | Forward | 5'-GTCACCCAACTCCAGGATTC-3' | | chr1:155,206,329-155,206,349 |
| GBA-F9 | Forward | 5'-CCTCACAGGGCTGACCTACC-3' | | chr1:155,205,728-155,205,748 |
| GBA-F10 | Forward | 5'-GAGCCTCTGCAGGAGTTATGG-3' | | chr1:155,205,188-155,205,209 |
| GBA-F11 | Forward | 5'-GGGCTTCCTGGAGACAATC-3' | | chr1:155,204,288-155,204,307 |

^a^ *GBA* gene (NCBI: NM_000157.4; GRCh37)

**Table S2**  *GBA* variants and its frequency in Thai GD (n = 68 alleles)

| Mutation change | Protein variant | | | Number of allele | | | | | % |
| --- | --- | --- | --- | --- | --- | --- | --- | --- | --- |
|  | 3 letter code | 1 letter code (traditional name) | present study | | other reference | | total | |  |
| \| c.115+1G>A \| \| --- \| | IVS2+1G>A | | | 4 | |  | | 4 | 5.8 |
| c.475C>T | p.Arg159Trp | p.R159W (R120W) | 1 | |  | | 1 | | 1.5 |
| \| c.681T>C \| \| --- \| | p.Asn227Lys | p.N227K (N188K) | 0 | | 1^a^ | | 1 | | 1.5 |
| c.754T>A | p.Phe252Ile | p.F252I (F213I) | 0 | | 2^b^ | | 2 | | 2.9 |
| c.762-1G>C | IVS6-1G>C | | | 2 | | 2^b^ | | 4 | 5.8 |
| c.913C>G | p.Pro305Ala | p.P305A (P266A) | 1 | |  | | 1 | | 1.5 |
| c.999+1G>A | IVS7+1G>A | | | 1 | |  | | 1 | 1.5 |
| c.1204T>C | p.Tyr402His | p.Y402H |  | | 1^a^ | | 1 | | 1.5 |
| c.1216A>G | p.Asn409Ser | p.N409S (N370S) | 1 | |  | | 1 | | 1.5 |
| c.1240G>C | p.Val414Leu | p.V414L (V375L) | 1 | |  | | 1 | | 1.5 |
| c.1389-3C>G | IVS9-3C>G | | | 1 | | 2^b^ | | 3 | 4.4 |
| c.1342G>C | p.Asp448His | p.D448H (D409H) | 1 | |  | | 1 | | 1.5 |
| c.1448T>C | p.Leu483Pro | p.L483P (L444P) | 33 | | 8^a,c^ | | 41 | | 60.3 |
| c.1609T>C | p.Ter537Ala | p.X537A | 0 | | 1^a^ | | 1 | | 1.5 |
| c.151C>T+ c.1599G>A (cis) | p.Ser384Phe +p.Trp533Ter | p.S384F+ p.W533*  (S345F+W484*) | 1 | |  | | 1 | | 1.5 |
| p.1448T>C+c.1483G>C+ c.1497G>C (cis) or Rec1a | Rec1a or [p.Leu483Pro+ p.Ala495Pro+ p.Val499Val] | Rec1a or [L444P+ A456P+ V460V] | 2 | |  | | 2 | | 2.9 |
| unidentified allele |  |  | 1 | | 1^a^ | | 2 | | 2.9 |
| total |  |  | 50 | | 18 | | 68 | | 100 |

^a^ Tammachote R et al., 2013 [17]; ^b^ Suwannarat P et al., 2007 [16]; ^c^ Chavananon S, et al, 2021 [15]

**Fig. S1** Nested PCR and locations of primers. A. Graphical methods of nested PCR and locations of primer used. B. Products of nested PCR and location of primers for sequencing.


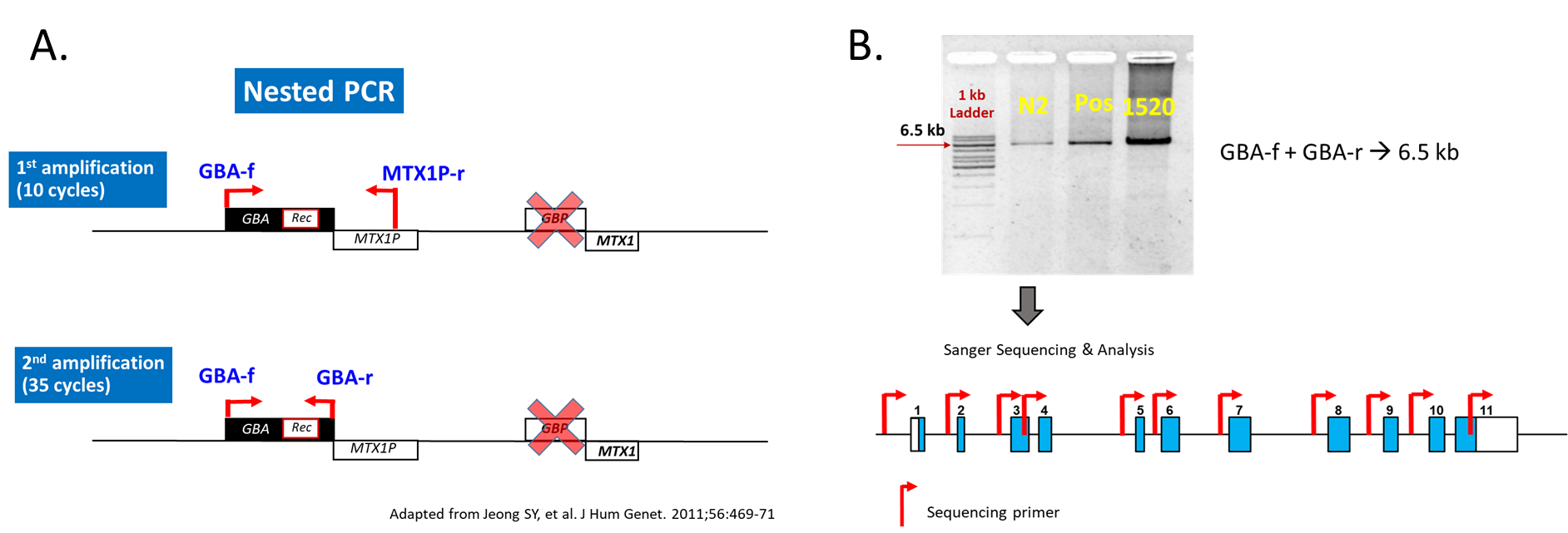


**Fig. S2**  Genomic organization of *GBA* and *GBAP* and sites of various recombination (gene conversion) alleles. Noted distinct sequences of *GBAP*.


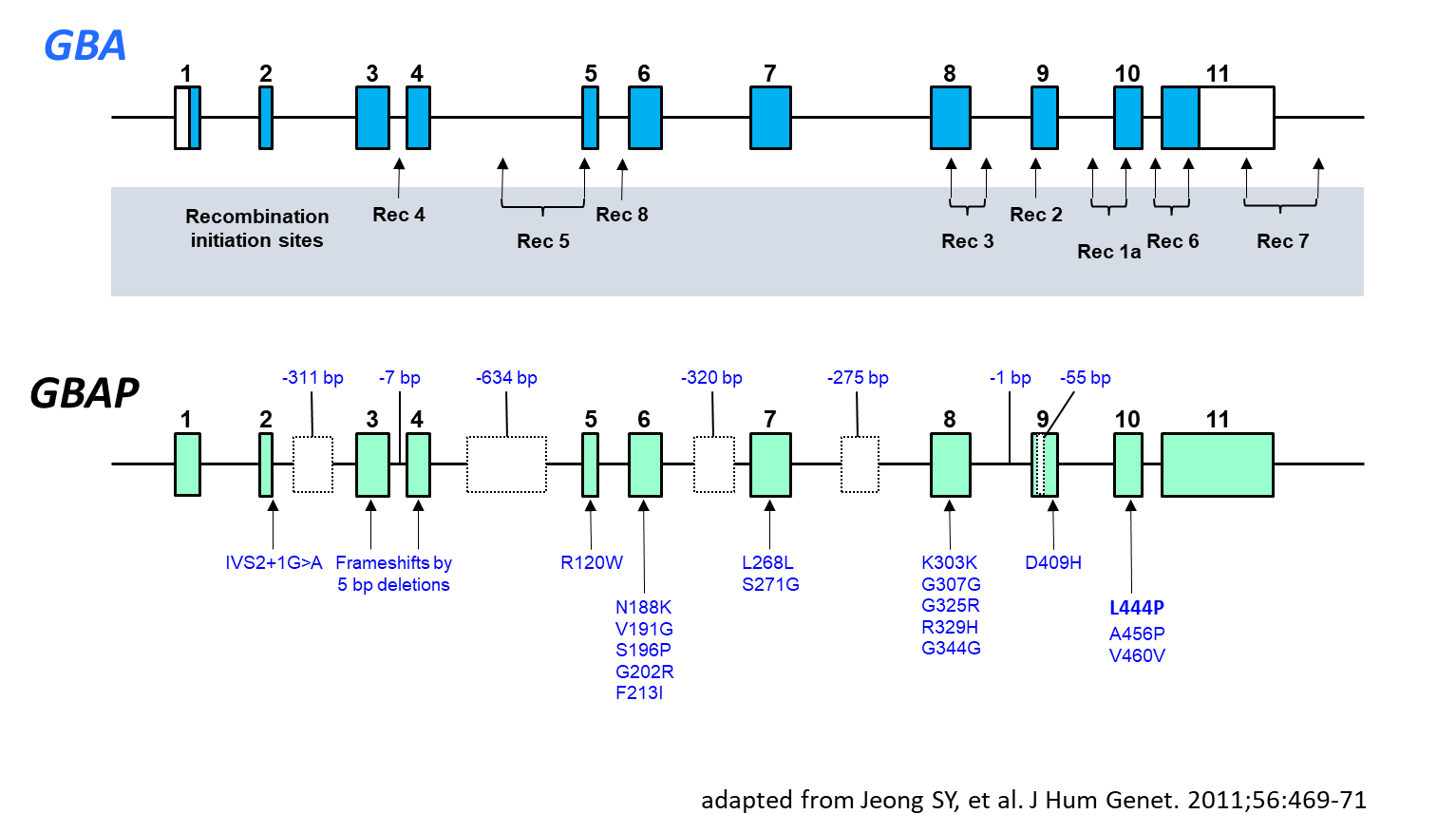


**Fig. S3**  Heterozygous gene conversion, Rec1a, allele. Noted 3 missense variants, p.L483L, p.A495P, p.V499V, on the same allele (in *cis*) indicating the Rec1a.


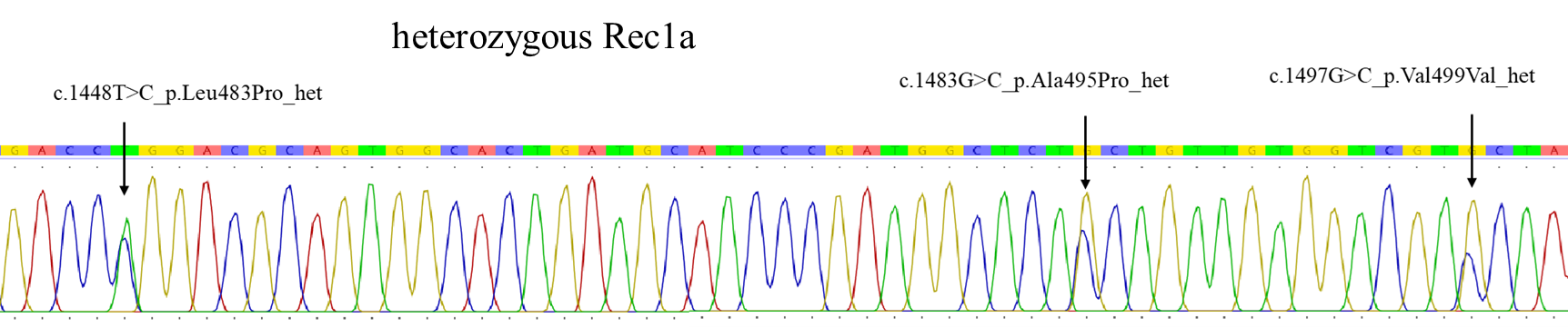


**Fig. S4** Prevalence of Gaucher disease phenotypes and *GBA* mutant alleles in total Thai patients. Data for the present study combined with three previous reports (total n = 36 for phenotype analysis, and 68 alleles for genotype analysis).


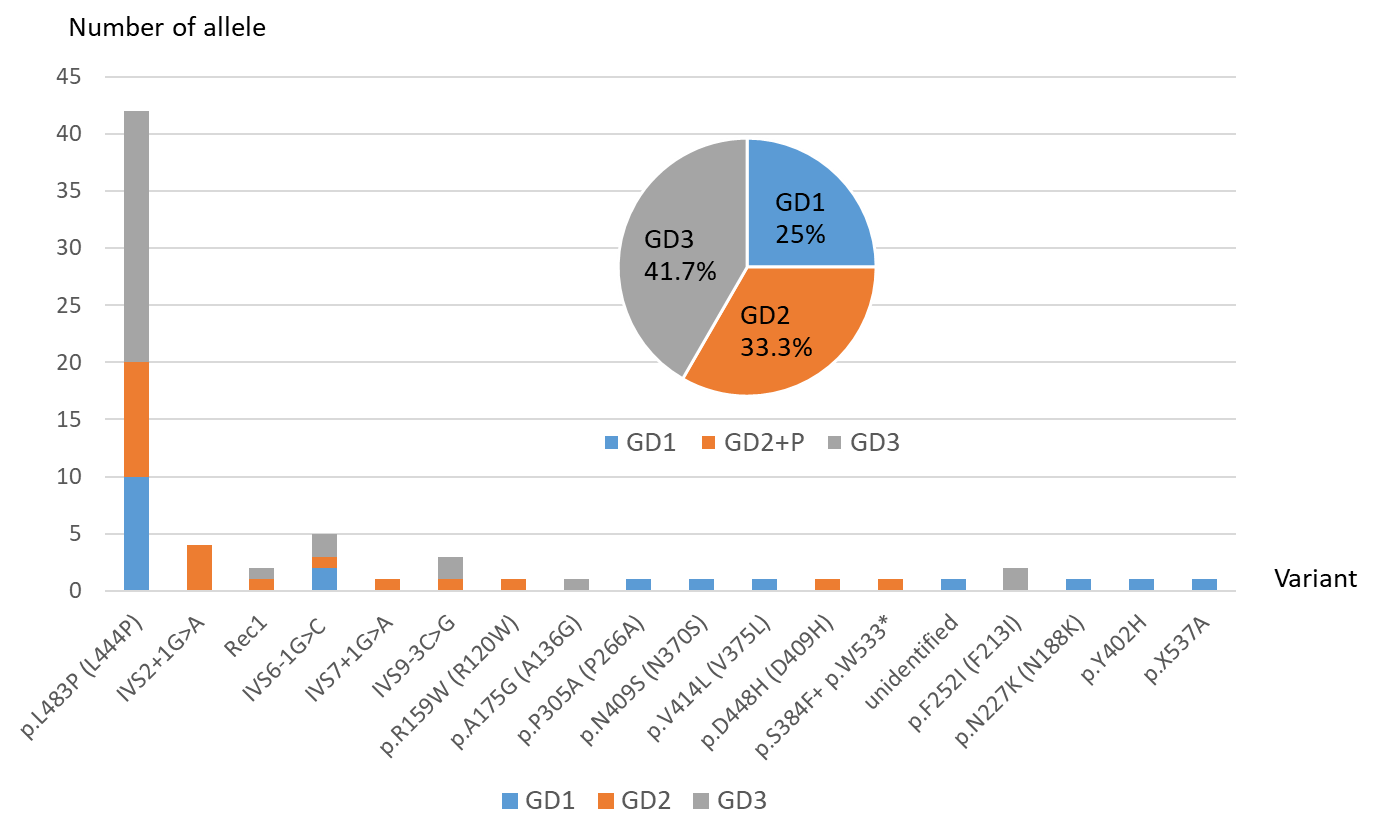


**List of References** (using the same running number as shown in the main text manuscript)

9. Jeong SY, Kim SJ, Yang JA, Hong JH, Lee SJ, Kim HJ. Identification of a novel recombinant mutation in Korean patients with Gaucher disease using a long-range PCR approach. J Hum Genet. 2011;56(6):469-71.

15. Chavananon S, Sripornsawan P, Songthawee N, Chotsampancharoen T. Successful Treatment of Gaucher Disease With Matched Sibling Hematopoietic Stem Cell Transplantation: A Case Report and Literature Review. J Pediatr Hematol Oncol. 2021;43(8):e1153-e5.

16. Suwannarat P, Keeratichamroen S, Wattanasirichaigoon D, Ngiwsara L, Cairns JR, Svasti J, et al. Molecular characterization of type 3 (neuronopathic) Gaucher disease in Thai patients. Blood Cells Mol Dis. 2007;39(3):348-52.

17. Tammachote R, Tongkobpetch S, Srichomthong C, Phipatthanananti K, Pungkanon S, Wattanasirichaigoon D, et al. A common and two novel GBA mutations in Thai patients with Gaucher disease. J Hum Genet. 2013;58(9):594-9.
